# Ambient AI Scribe Implementation in an Ambulatory Setting in a Single Medical Group: Prospective Study

**DOI:** 10.2196/84104

**Published:** 2026-06-23

**Authors:** Cameron J Harvey, Josiah Morita, William Huynh, Russell K Woo, Jerome P Lee

**Affiliations:** 1 Tripler Army Medical Center, 1 Jarrett While Rd, Honolulu, HI, 96859, United States, 1 2172943329; 2 The George Washington University, Washington D.C., DC, United States; 3 Hawaii Pacific Health Medical Group, Honolulu, HI, United States; 4 Kapiolani Medical Center for Women and Children, Honolulu, HI, United States

**Keywords:** ambient artificial intelligence, documentation, burnout, work satisfaction, efficiency, health care technology, artificial intelligence, AI

## Abstract

**Background:**

Health care providers spend an excessive amount of time within electronic medical record (EMR) systems documenting patient encounters, often amounting to hours of work outside of regular office hours. This affects physician productivity and directly contributes to burnout. Artificial intelligence (AI) is becoming more integrated into medical care, including the development of speech recognition and note generation algorithms. Limited studies exist on how these AI tools affect provider satisfaction, work-life balance, and patient satisfaction.

**Objective:**

The aim of this study was to assess the use of ambient AI in medical documentation and its effects on time spent in the EMR and on provider burnout, with a secondary focus on note quality and patient satisfaction.

**Methods:**

This prospective study was conducted at the Hawaii Pacific Health Medical Group to pilot an AI note writer. Abridge was chosen as the AI platform and integrated with the Epic EMR. A goal of 75 providers for a 3-month pilot period was established from December 2024 through February 2025. Surveys were distributed to providers before and during the trial period. Epic Signal and Abridge data were used to correlate provider-perceived outcomes with EMR-recorded outcomes. Users were then divided into groups based on frequency of AI use, with high use defined as ≥60% AI scribe use in patient encounters. The primary outcome was time spent on documentation per appointment.

**Results:**

A total of 80 providers were recruited, with 79 completing the pilot. More than 25,000 notes were generated across 23 specialties. Signal metrics found a 21% decrease in time spent on notes per day (−13.6 minutes) and a 13% decrease in pajama time (−3.6 minutes) among high users. Among 79 providers using ambient AI, 6 (7.6%) reported spending ≥8 hours per week on notes outside of clinic hours, a 76% decrease from 25 (31.6%) providers before the pilot. With ambient AI, 39 (49.4%) physicians reported no burnout symptoms, representing a 22% increase. Provider-perceived workload decreased, and self-reported note quality remained favorable. Providers reported that 84% of notes required edits to less than one-quarter of the note content. Patient experience, as measured by “provider listened to me” scores on patient satisfaction surveys, was not significantly affected by ambient AI use (*P*=.39).

**Conclusions:**

This ambient AI scribe decreased the time providers spent writing notes in the clinic and decreased time spent in the EMR outside of work hours. There was no significant difference in symptoms of burnout.

## Introduction

Physician workload and burnout have been directly tied to several factors, with one of the chief factors being time spent documenting in the electronic medical record (EMR) [1]. Burnout has been an increasing problem for physicians, and the EMR is closely tied to this, with 75% of providers identifying the EMR as a contributor to burnout symptoms [2]. This factor has recently become potentially modifiable with the influx of ambient artificial intelligence (AI) scribes in the market. These ambient AI scribes are part of a growing body of AI that uses large language models (LLMs) to train algorithms. LLMs train AI using large volumes of data and typically function to generate outputs (eg, text) in response to prompts, and they have generated much excitement around potential applications in health care [3].

Ambient AI scribes have a goal of reducing physician workload in the EMR while maintaining high standards of robust clinical documentation. Current literature surrounding ambient AI scribes in medical documentation shows that they reduce the amount of time physicians spend writing notes outside of work hours, with a perceived effect on time management [4,5]. The effect size of ambient AI scribe use on time savings and provider satisfaction has been shown to be most prominent among high users; however, the exact definition of a high user is not standardized, ranging from AI scribe use in 60% to 70% of notes across most studies [4,6,7].

AI scribes are being quickly and widely implemented across institutions in the United States; however, data on their outcomes are limited to a few single institutions, with most studies including small numbers of providers and lacking correlation with other important outcomes, including patient experience. There may also exist institutional differences in culture that change the effect of AI scribe implementation, which may only be uncovered with further institutional implementation and review. Additionally, there are many options for ambient AI scribes in the market, and their algorithms and capabilities are evolving rapidly, making the continued reporting of outcomes critical to this growing body of literature. This study sought to define how implementation of the Abridge ambient AI scribe at a single medical group impacted provider time in the EMR and how this technology affected self-reported symptoms of burnout, note quality, and patient satisfaction scores.

## Methods

### Setting and Selection of Participants

Participants were selected from the Hawaii Pacific Health Medical Group for this pilot program, which was conducted in an ambulatory setting. This medical group provides care across the islands of Hawaii and includes 4 hospitals and 70 clinics located statewide. A minimum participation of 75 providers was determined per agreement between the medical group and Abridge for a 3-month trial period. All selected providers attended an instructional session and completed a prepilot survey before they were allowed to use the AI tool. Any provider not meeting these prerequisites was dropped from the program and replaced by another provider. A total of 80 providers were recruited and met the prerequisites to begin use of the AI tool in November 2024. After a familiarization and onboarding period in November, the 3-month pilot program was conducted from December 1, 2024, through February 28, 2025.

### Ethical Considerations

This study (2024-080) was reviewed by Hawaii Pacific Health Research Institute officials and determined to be exempt from the institutional review board’s review, as it was determined to fall under a quality improvement initiative. Participants in this study were physicians who volunteered and agreed to participate. Physicians were not required to provide written informed consent to participate. After careful ethics review and consideration by Hawaii Pacific Health, it was decided that use of this ambient AI technology during health care visits would require verbal consent from patients prior to use, and this consent was documented in the encounter note. Patient refusals were not recorded as part of this study. Per the ambient AI company, recordings and transcripts are stored on secure HIPAA (Health Insurance Portability and Accountability Act)−compliant servers for 30 days before deletion. Notes were self-audited by providers prior to submission to the medical record to ensure that there were no inaccuracies generated by the ambient AI scribe in official clinical documentation. The research team deidentified data for distribution and publication.

### Data Collection

The conceptual framework for this study for data collection and analysis was primarily to evaluate time-saving capability, followed by burnout and workload demands. We sought to measure time savings using self-reported time in the EMR and relate those findings to actual time in the EMR using Epic Signal data. This information was then used to better understand self-reported workload demand and burnout survey results.

Data from the familiarization period were excluded from analysis as providers were added to the pilot and allowed time to learn the AI model. Safety of the AI-generated notes was determined by provider self-auditing of notes and was assessed using 5-point Likert scale responses and free-text responses from providers. To measure provider-perceived effectiveness of the AI tool, another survey with Likert scale scores was sent before and after the pilot (survey example shown in Multimedia Appendix 1). Surveys were not anonymous and were used to match provider responses from prepilot and postpilot results. Questions derived from the National Aeronautics and Space Administration–Task Load Index (NASA-TLX) scale were scored on a 21-point scale (0‐20). The rest of the survey questions were scored on a 5-point scale, including burnout and perceived ability to provide undivided attention to patients. The survey also included a self-reported measure of time spent in the EMR outside office hours per week, with response options provided in 1-hour intervals ranging from less than 1 hour to more than 10 hours. All providers who were included in the study completed all surveys, totaling 79 surveys for each of the prepilot and postpilot periods. Provider charting metrics, including “pajama time,” time in notes per appointment and per day, and number of characters used per note, were collected from Epic Signal. Epic defines pajama time as the average number of minutes per scheduled day spent on charting activities outside of 7 AM to 5:30 PM on weekdays, time outside scheduled hours on weekends, and time on unscheduled holidays. Patient satisfaction data for the proportion of patients responding “Yes, definitely” to the question, “Did this provider listen to you carefully?” on patient satisfaction surveys were collected from optional patient surveys and matched to each provider. Ambient AI scribe use metrics were collected and provided by Abridge. High use was defined as use of the ambient AI scribe on at least 60% of notes, in conformity with previously reported definitions [6]. An example of the survey for the precollection and postcollection periods is included in Multimedia Appendix 1.

### Statistical Analyses

For simple outcomes, descriptive statistics were used. For categorical data, including burnout rates, patient satisfaction, and provider-reported work hours outside of normal office hours, a chi-square test of association was used, and odds ratios (ORs) were calculated to test significant associations. Continuous variables, including Epic Signal metrics for time in notes per appointment, time in notes per day, pajama time, and progress note length, were compared with a paired 2-tailed Student *t* test. Likert scale questions were averaged for each group and compared with a paired 2-tailed Student *t* test, the results of which are displayed with a Gardner-Altman estimation plot to show effect size. To confirm internal consistency of the NASA-TLX data, Cronbach alpha was calculated and found to be 0.77, indicating acceptable internal consistency. A frequency distribution graph was created for all participants and fitted with a Gaussian distribution curve. Goodness of fit was determined by an *R*^2^ value. A graph detailing use by specialty was created by graphing mean use with SE of the mean (SEM) for all specialties with ≥3 participants. Between-group use was then compared using a Brown-Forsythe ANOVA test with a post hoc Dunnett T3 multiple comparison test to compare each group. GraphPad Prism (version 10.6.1; GraphPad) was used for all statistical tests and graph creation.

## Results

**Figure 1.**
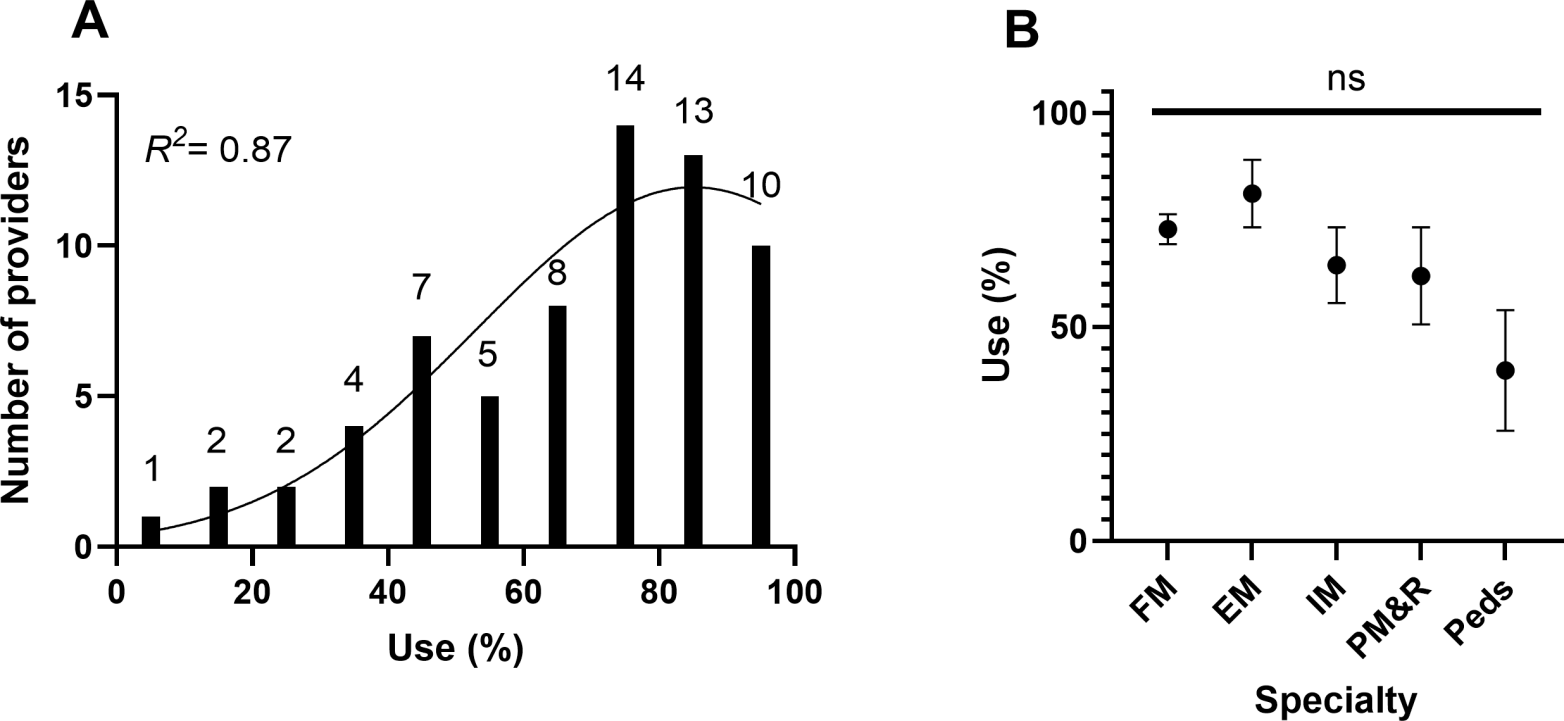
Ambient artificial intelligence scribe use among participating providers. (A) Frequency distribution of use among all pilot users. (B) Use by specialty for specialties with >3 users. EM: emergency medicine; FM: family medicine; IM: internal medicine; ns: no significance; Peds: pediatrics; PM&R: physical medicine and rehabilitation.

A total of 80 providers were recruited for this pilot study, and 79 (99%) providers completed the entire 3-month study period. Over this pilot period, more than 25,600 notes were generated and included in the analysis. Of the 79 providers that completed the pilot, 45 (57%) were in the high-user group, maintaining monthly use of Abridge in ≥60% of notes. There were 4 (5%) providers who used the AI on more than 1000 notes, with the highest-volume user generating 1415 notes in an urgent care setting. A total of 23 specialties were represented, with most from primary care, including 24 (30%) providers from family medicine and 12 (15%) from internal medicine. A frequency distribution fitted with a Gaussian curve is shown in Figure 1A, fit with a Gaussian curve. An *R*^2^ goodness-of-fit test for the curve was 0.87, indicating a good fit. Average use by specialty with ≥3 participants was calculated for family medicine (mean 72.85%, SD 16.82%), internal medicine (mean 64.44%, SD 27.94%), emergency medicine (mean 81.16%, SD 19.39%), physical medicine and rehabilitation (mean 61.94%, SD 19.61%), and pediatrics (mean 39.84%, SD 24.39%; Figure 1B). An ANOVA did not find any difference in mean outcomes, and a post hoc multiple comparisons test did not find a significant difference between any groups (*P*=.13).

**Figure 2.**
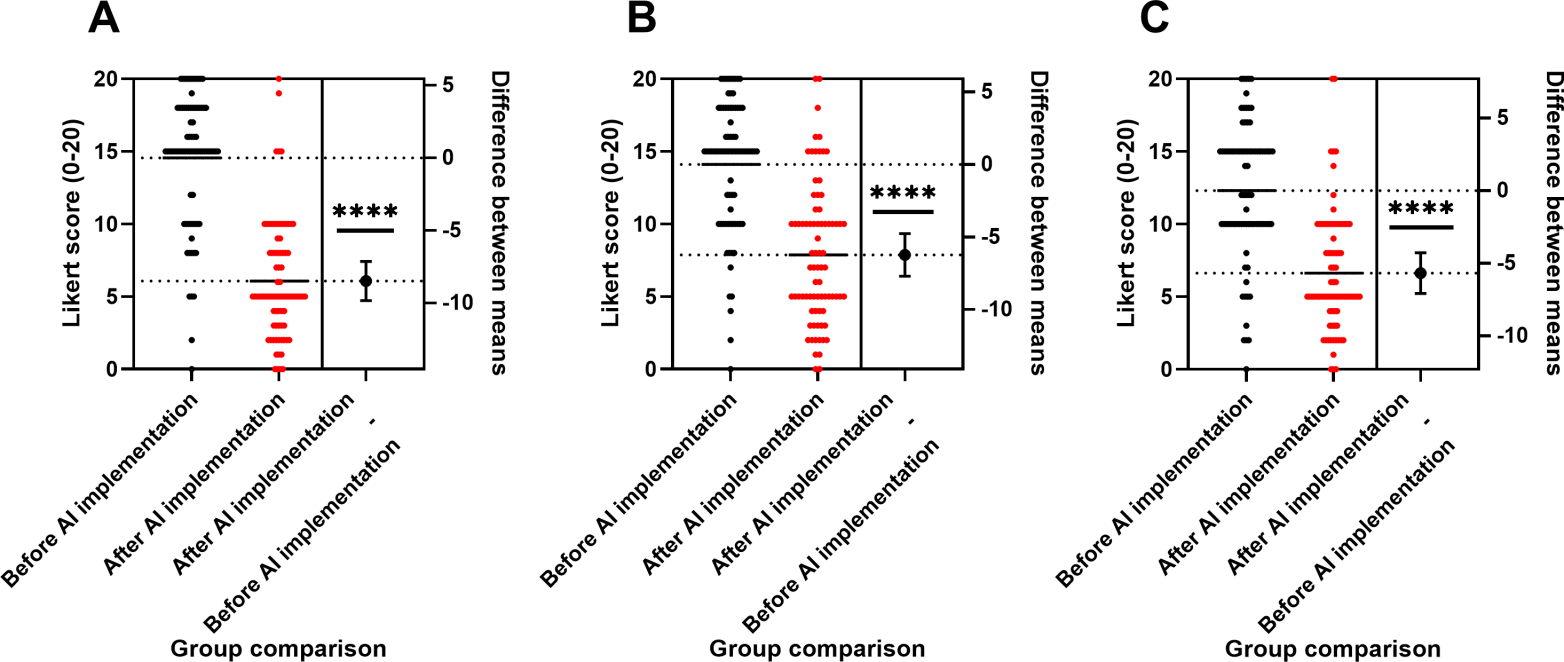
Provider task load questionnaire. In preimplementation and postimplementation surveys, providers were asked questions derived from the validated National Aeronautics and Space Administration–Task Load Index on workload perception. Results were shown for (A) “How rushed are you?” (B) “How hard do you have to work?” and (C) “How mentally demanding is writing notes?” AI: artificial intelligence. ****P<.001.

Provider-perceived outcomes for workload reduction using the NASA-TLX survey were quantified with pre-AI and post-AI surveys using Likert scale scoring for all 79 participants. A scale of 0 to 20 was used to assess providers’ perceptions of mental demand, time demand, and effort. The average score for mental demand decreased by 46% (12.3 pre-AI score to 6.6 post-AI score; *P*<.001), time demand decreased by 58% (14.6 pre-AI score to 6.1 post-AI score; *P*<.001), and effort decreased by 44% (14.1 pre-AI score to 7.9 post-AI score; *P*<.001; Figure 2).

Provider self-reported symptoms of burnout were tabulated on a 5-point Likert scale, with responses of ≥3 indicating perceived burnout. Reported burnout declined by 15% from 47 (59.5%) to 40 (50.6%) respondents after using Abridge for 3 months; however, the change in proportions was not statistically significant (OR 1.4, 95% CI 0.8-2.7; *P*=.34; Figure 2). There was a 3% decrease (from n=3, 3.8% providers to n=1, 1.3% provider) in scores of 5, “I feel completely burned out. I am at the point where I may need to seek help.” The average Likert score decreased from 2.8 to 2.6, but this difference was not statistically significant (*P*=.18).

**Figure 3.**
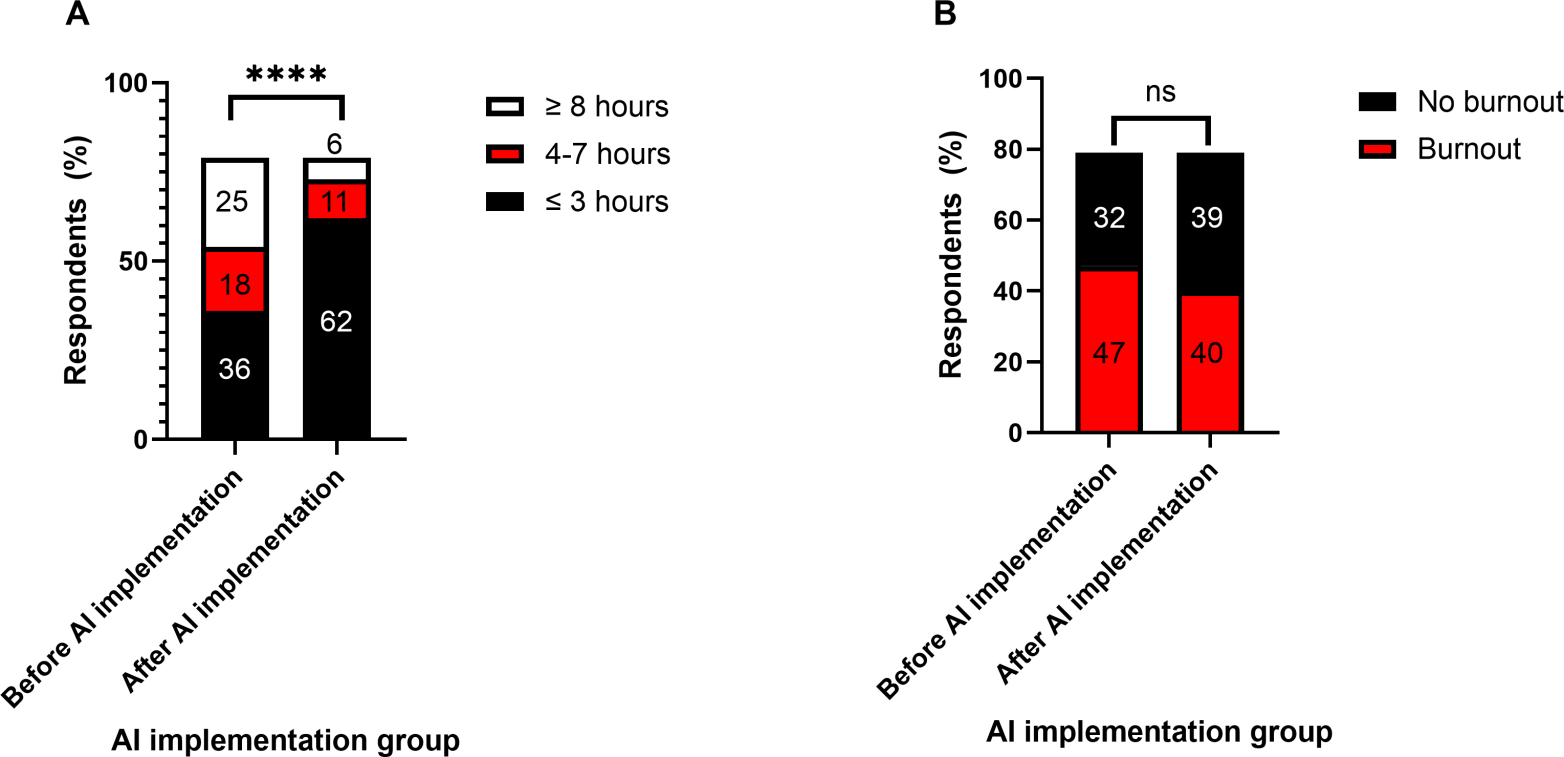
Provider self-reported survey responses for (A) time spent in the electronic medical record outside of clinic hours and (B) burnout symptoms before and after implementation of an ambient artificial intelligence (AI) scribe. Absolute values for respondents are placed within the bars. ****P<.001. ns: no significance.

Self-reported time in the EMR outside of clinic hours was also assessed on an hours-per-week basis. In the pre-AI survey, 25 (32%) providers reported working in the EMR outside of the clinic ≥8 hours per week, 18 (23%) worked 4-7 hours per week, and 36 (46%) worked ≤3 hours per week (Figure 3). In the post-AI survey, the number of providers reporting working ≥8 hours per week in the EMR outside clinic hours decreased to 8% (n=6), while those reporting 4 to 7 hours per week decreased to 14% (n=11). A total of 62 (78%) respondents reported spending ≤3 hours in the EMR outside of the clinic per week after implementing the AI tool. The difference between the pre-AI and post-AI groups was statistically significant (*P*<.001).

Next, Epic Signal data were used to quantify objective changes in provider note creation and output among high users (AI used on ≥60% of notes). The average time in notes per appointment decreased from 7.14 minutes to 5.63 minutes (21% decrease; *P*=.005), while the average time in notes per day decreased by 21% (*P*<.001; Table 1). Pajama time decreased from 27.47 minutes per day on average to 23.89 minutes per day, a 13% decrease that was not significant (*P*=.19). There was an increase in the average number of characters per note by 7%, from 5741 characters to 6121 (*P*=.001).

**Table 1.** Epic chart use changes among high users.

Metrics	Baseline, mean (SD)	Pilot period, mean (SD)	Percentage change	95% CI of the difference in means	*P* value
Time in notes per appointment (minutes)	7.14 (3.54)	5.63 (3.50)	−21	−2.07 to −0.37	.005
Time in notes per day (minutes)	66.24 (37.04)	52.63 (31.21)	−21	−18.18 to −8.35	<.001
Pajama time (minutes)	27.47 (35.72)	23.89 (24.96)	−13	−5.77 to 1.20	.19
Progress note length (characters)	5741 (2433)	6121 (2615)	7	234 to 898	.001

Provider satisfaction with note quality was assessed using a 5-point Likert scale survey evaluating accuracy (eg, no hallucinations), thoroughness (eg, no critical omissions), appropriateness of tone, and the proportion of the note that required editing. There were 494 notes that were analyzed for safety by this method. Favorable ratings (responses of 4 or 5) were high with accuracy at 86.6% (n=428), thoroughness at 82.5% (n=408), and appropriate tone at 90.3% (n=446; Table 2). Most of the AI-generated notes required little editing, with providers reporting that 83.6% (n=413) of notes required editing of less than one-quarter of the note, and 21.1% (n=104) of notes did not require any editing by the provider prior to submitting the note to the EMR. Only 13 (2.6%) of notes required editing of at least 75% of the note while 26 (5.3%) required 51%-75% of the note to be edited.

**Table 2.** Results of provider self-audited surveys assessing note quality and safety.

Responses	Accuracy, n (%)	Thoroughness, n (%)	Appropriate tone, n (%)
Not at all	12 (2.4)	14 (2.8)	13 (2.6)
(2)	11 (2.2)	13 (2.6)	6 (1.2)
Somewhat	53 (10.7)	68 (13.8)	38 (7.7)
(4)	218 (44.1)	198 (40.1)	149 (30.2)
Extremely	200 (40.5)	201 (40.7)	288 (58.3)

Patient satisfaction scores for “provider listened to me” were collected for each participating provider. A total of 3393 surveys were collected for high users during the prepilot period, and 2796 surveys were collected during the pilot period. Among all providers, the proportion of patients responding “yes, definitely” did not change. Among high users there was a nonsignificant increase in this score from 92.6% to 93.2% (OR 0.91, 95% CI 0.75-1.1; *P*=.36). In the pre-AI and post-AI surveys, there was a 17% increase in the mean Likert score (3.5 to 4.1 out of 5) for providers reporting that they were able to give undivided attention to patients during visits (*P*<.001).

## Discussion

### Principal Findings

In this pilot study conducted within a single medical group spanning multiple medical centers and clinic sites, we found that physicians who used the ambient AI scribe experienced an average reduction in time spent on notes and in the EMR. Not only did providers perceive improvements in EMR charting time, but they also reported decreased workload pertaining to clinic note-writing responsibilities. Despite these changes, there was no significant change in the distribution of symptoms of burnout by the end of the pilot period. Use among all participants was found to fit a normal Gaussian distribution, and there was no difference in use between specialties. Although high use was encouraged, 57% (45/79) of providers met high-user status.

Although we did find decreases in time spent within the EMR and on writing notes based on Epic Signal data, this time saving was relatively small, averaging only 13.6 minutes saved per day in note writing. This finding differs from the self-reported time spent in the EMR outside of clinic hours, where most providers reported hours of time saved per week after using the ambient AI scribe. This could represent an error in judgment among providers given relative differences in individual interpretation. Interpretation of this discrepancy is further limited by the inclusion of all participating providers in survey analyses, while only high users were included in the analysis of Signal metrics. This may also point to the usefulness of an ambient AI scribe as a mental offloading tool, where, despite less time saved than perceived, there is still a benefit for providers. This coincides with NASA-TLX data, where the validated index for mental workload found significant decreases in perceived workload on average [8].

This reduction in mental workload did not directly translate into reduced burnout, as the change in burnout was not statistically significant. Burnout is not reliably reduced by ambient AI scribes in studies thus far. Our study used a coarse, nonvalidated measure of burnout, which limits interpretation and is prone to bias. However, ongoing studies seek to use validated burnout metrics and to better understand the relationship between perceived mental workload reduction and burnout outcomes.

### Comparison to Prior Literature

Previous literature on ambient AI scribes has been mixed in terms of outcomes and efficacy. The largest study to date, conducted by Kaiser Permanente in California, was published in 2024 and reported the results of a pilot program using Abridge among approximately 1000 providers [4]. Although this study is large, the authors performed analyses on all providers who generated at least 100 notes with the ambient AI scribe and did not stratify results by use status, thereby limiting direct comparison with our data. Despite this limitation in direct comparison, their study also found decreased time spent in the EMR and noted overall favorable remarks from physicians and patients alike.

One major limitation in the literature is the lack of data on long-term use, which these Kaiser Permanente authors recently published in a 1-year follow-up study [9]. The group found 15,700 hours of time saved in clinical documentation for AI scribe users compared to nonusers over the study year, with continued uptake and use, especially among high users, whom they define as the top third of users. Further long-term data collection will be necessary to examine the maintenance of time savings and the potential effects on loss of participants through attrition.

As this scribe is released to more providers, it can be anticipated to aid in increasing provider satisfaction, retention, and longevity, all of which have been declining critically short nationwide [10]. How ambient AI scribes affect burnout is also contested, with some studies finding significant improvements while others, including this study, have found no significant difference [11-14]. Although benefits for burnout remain contested, perceived reductions in workload are consistent across studies and are in agreement with our findings [11,12]. Additionally, subgroup analyses of outcomes for different specialties remain limited, especially outside the realm of primary care [15].

### Safety and Patient Experience

A secondary outcome of this study was to determine the safety of this ambient AI scribe to ensure that patients and physicians are protected. Providers considered the ambient AI scribe safe to use for their patients, as long as there was examination of each note prior to submission, because, although infrequent, hallucinations and omissions occurred. Ensuring that notes encompass the critical visit details without omissions or hallucinations is a critical metric for all stakeholders. Our study used provider self-auditing and self-reporting on random notes to examine the safety profile. Independent validation of ambient AI safety is difficult because the underlying development of LLM algorithms is proprietary and varies between products, so we must rely on output metrics. Other studies have attempted to define the safety of these products but largely reach similar conclusions: even when these tools are effective, there are noticeable rates of errors, with the most common being omissions [16-18].

Patient satisfaction is also imperative to assess during this rollout to ensure that the introduction of new technology does not hinder or negatively affect the patient-physician interaction. Our results showed no change in patient satisfaction scores for providers participating in the pilot. Although we found that high users had a nonsignificant increase in the “provider listened to me” score on patient surveys, more data collection over time with longer implementation will be more useful for the interpretation of this effect. Other literature on patient perception of the impact of AI scribes on clinic visits is limited. One study surveying 21 patients at a single clinic found that 71% of patients said they felt they spent more time with their physician, while only one patient said they spent less time [4].

### Limitations

There are several limitations to this study. First, we rely, for several metrics, on self-reported survey responses, which are prone to bias. Although we attempted to substantiate some self-reported responses with objective measures, such as time spent in notes outside of work, this could not be accomplished for all metrics. This is particularly true of burnout. Second, self-auditing notes for safety are similarly prone to bias. This form of note evaluation is suboptimal for an in-depth analysis of note-writing capacity for this ambient AI. However, we did not include more thorough objective measures of note-writing analysis as this was not the primary objective of the study, and this area has already been studied for this technology platform [18,19]. Third, despite encouragement to participate, only 45 of the 79 participants met high-user status by using the ambient AI scribe in at least 60% of notes. This decreases the power of the study and points to barriers to uptake that are multifactorial and should be subject to further study.

### Future Directions

The findings presented here offer opportunities for further study on ambient AI in the clinical workspace. One opportunity is to expand the cohort to increase the power of the study. By increasing the number of participants, we will also increase the representation of different specialties for subgroup analyses to better understand whether this technology is better suited to specific roles. Expanded power and use of a validated instrument for assessment of burnout will aid in the analysis of the interplay between workload reduction and burnout outcomes [20]. Furthermore, recording and analyzing patient refusals of ambient AI scribe use during encounters, as well as the reasons for those refusals, may elucidate a better understanding of how incorporation and patient education can be improved. Next, further analysis of outcomes by use group (high user vs low user) would improve understanding of the application of this technology and its association with work-life benefit outcomes. Additionally, our providers noted limitations with non-English speaking encounters where there may be an increased occurrence of errors. Noted problems with non-English encounters include the difficulty of the ambient AI scribe picking up contextual exchanges, translating details, and reducing the need for extensive editing in non-English languages. Further study of limitations in these scenarios will aid in improved product development and application to patient care. Finally, ambient AI scribes represent a rapidly evolving landscape with continuous changes and improvements across platforms and interfaces. These changes are anticipated to enhance usability while improving workflow. For example, queuing orders captured during discussion and recommending appropriate evaluation and management service levels are planned integrations that could improve time-saving capability, reduce mental workload, and mitigate burnout, especially since poor EMR usability has been tied to burnout [10].

### Conclusions

This pilot study of 79 providers in a single medical group evaluated the implementation of the Abridge ambient AI scribe and found a decrease in note-writing time in the clinic. There was no significant difference in reported burnout symptoms, although self-reported workload did decrease.

## Supplementary material

10.2196/84104 Multimedia Appendix 1 Example of the prepilot and postpilot survey questionnaire sent to providers.

## Abbreviations

AI: artificial intelligence
EMR: electronic medical record
HIPAA: Health Insurance Portability and Accountability Act
LLM: large language model
NASA-TLX: National Aeronautics and Space Administration–Task Load Index
OR: odds ratio
