# Supplementary figures and images for: Ambient AI Scribe Implementation in an Ambulatory Setting in a Single Medical Group: Prospective Study
Source: JMIR Med Inform. 2026 Jun 23;14:e84104. doi: 10.2196/84104 (PMC13289844; doi:10.2196/84104)

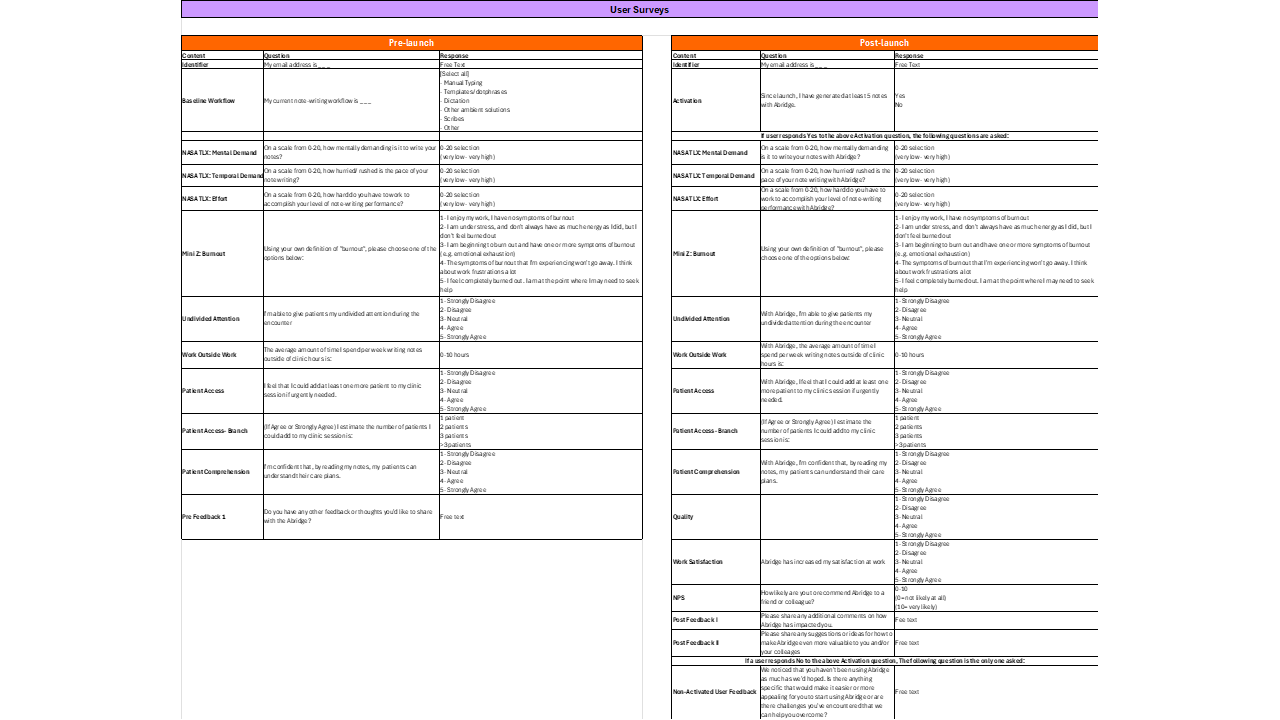

Supplement: Multimedia Appendix 1 [file medinform-v14-e84104-s001.png]
